# Interaction of the Deubiquitinating Enzyme Ubp2 and the E3 Ligase Rsp5 Is Required for Transporter/Receptor Sorting in the Multivesicular Body Pathway

**DOI:** 10.1371/journal.pone.0004259

**Published:** 2009-01-23

**Authors:** Mandy H. Y. Lam, Danièle Urban-Grimal, Amandine Bugnicourt, Jack F. Greenblatt, Rosine Haguenauer-Tsapis, Andrew Emili

**Affiliations:** 1 Banting and Best Department of Medical Research, Donnelly Centre for Cellular and Biomolecular Research, Department of Molecular Genetics, University of Toronto, Toronto, Ontario, Canada; 2 Institut Jacques Monod-CNRS, Universités Paris VI and Paris VII, Paris, France; The University of Queensland, Australia

## Abstract

Protein ubiquitination is essential for many events linked to intracellular protein trafficking. We sought to elucidate the possible involvement of the *S. cerevisiae* deubiquitinating enzyme Ubp2 in transporter and receptor trafficking after we (this study) and others established that affinity purified Ubp2 interacts stably with the E3 ubiquitin ligase Rsp5 and the (ubiquitin associated) UBA domain containing protein Rup1. *UBP2* interacts genetically with *RSP5*, while Rup1 facilitates the tethering of Ubp2 to Rsp5 via a PPPSY motif. Using the uracil permease Fur4 as a model reporter system, we establish a role for Ubp2 in membrane protein turnover. Similar to hypomorphic *rsp5* alleles, cells deleted for *UBP2* exhibited a temporal stabilization of Fur4 at the plasma membrane, indicative of perturbed protein trafficking. This defect was ubiquitin dependent, as a Fur4 N-terminal ubiquitin fusion construct bypassed the block and restored sorting in the mutant. Moreover, the defect was absent in conditions where recycling was absent, implicating Ubp2 in sorting at the multivesicular body. Taken together, our data suggest a previously overlooked role for Ubp2 as a positive regulator of Rsp5-mediated membrane protein trafficking subsequent to endocytosis.

## Introduction

Protein ubiquitination is essential for the proper functioning of many eukaryotic cellular processes. While the covalent conjugation of polyubiquitin chains to a protein by ubiquitin ligases often leads to subsequent targeting and degradation by the 26S proteasome [1], ubiquitin attachment has also been shown to be critical for the correct execution of non-proteasomal events, ranging from the regulated methylation of histones during transcriptional elongation [2] to the intracellular trafficking of proteins [3]. For the latter, ubiquitination serves as a key signal mediating the internalization, intracellular transport and subsequent recycling or vacuolar degradation of plasma membrane-bound receptors and transporters [4]. While much progress has been made in elucidating the mechanistic basis for various steps in protein trafficking, many aspects about the core components and enzymatic steps remain unresolved.

In the budding yeast *Saccharomyces cerevisiae*, a single essential E3 ubiquitin ligase, Rsp5, has been implicated in the internalization of most if not all endocytosed proteins [5]. The uracil permease Fur4 is an experimentally well-characterized model substrate of Rsp5. Uracil binding, or various stress conditions such as nutrient starvation, heat shock, or the inhibition of protein synthesis trigger Fur4 phosphorylation which drives the subsequent recognition and Lys63-linked di-ubiquitination of Fur4 by Rsp5 on two specific lysine residues [6]–[10]. Similar to other membrane proteins, the ubiquitination of Fur4 results in its rapid and complete clearance from the plasma membrane into early endosomes. This is followed by the subsequent recognition of ubiquitinated Fur4 by the ESCRT-I, II, III complexes and other class E Vps proteins, followed by sorting into multivesicular bodies (MVBs) [11]. When MVB sorting is inhibited in class E *vps* mutants, endocytosed Fur4 recycles back up to the plasma membrane [12]. After sorting into the MVB, Fur4 is targeted to the vacuolar lumen where it is degraded [10]. Fur4 can also follow a different pathway when synthesized in the presence of its substrate, uracil. In this situation, newly synthesized Fur4 is diverted to the endosomal system directly from the Golgi apparatus [13]. It then undergoes Rsp5-dependent ubiquitination, a modification crucial for its sorting into MVBs [14], followed by vacuolar degradation after fusion of MVBs with the vacuole.

The process by which ubiquitin chains are attached to proteins is reversible. Cleavage of ubiquitin chains from ubiquitinated proteins is performed by a broad family of deubiquitinating enzymes (DUBs) which share conserved motifs, notably the Cys and His boxes, which are required for catalytic function [15]. In yeast, these DUBs fall into two major categories [16]: the ubiquitin C-terminal hydrolases (Uch), and the ubiquitin specific processing proteases (Ubp). Uchs are restricted to cleaving ubiquitin from small peptides and chemical adducts, while Ubps release ubiquitin from larger protein substrates and disassemble longer polyubiquitin chains [1]. Based on sequence analysis, the budding yeast *S. cerevisiae* encodes 16 Ubps, most of which have been confirmed to have general DUB activity *in vitro* [17].

Emerging evidence implicate members of the vesicle-based intra-organellar protein trafficking pathway as physiologically relevant targets of several of the Ubps. These include: Doa4 (Ubp4), involved in the deubiquitination of internalized endocytic cargoes such as Fur4 at the endosome immediately prior to their sorting into MVBs [18]; Ubp3, implicated in the deubiquitination of regulatory proteins in both the anterograde [19] and retrograde [20] protein transport pathways through the endoplasmic reticulum (ER) and Golgi systems, along with a possible involvement in the cytoplasm to vacuole (Cvt) trafficking pathway [21]; and Ubp1, which has been linked to the internalization and turnover of the ABC membrane transporter Ste6 via an as yet unknown mechanism [22].

Parallel studies by our group (described in this study) and another laboratory [23] have now established specific and physiologically significant physical and functional interactions between the *S. cerevisiae* deubiquitinating enzyme Ubp2, the E3 ligase Rsp5, along with a protein of previously unknown function, Rup1, which contains a ubiquitin associated (UBA) domain and a putative Rsp5-binding motif. Although the *in vitro* assays reported previously [23] have hinted at a possible role of Ubp2 in the deubiquitination of substrates of Rsp5, including the ER membrane bound transcription factor Spt23 and the two relatively uncharacterized proteins Csr2 and Ecm2 [24], physiologically significant targets of Ubp2 with respect to Rsp5-mediated trafficking remains uncertain. Here, we expand upon these initial observations and establish a critical role for Ubp2 in the ubiquitin-dependent sorting of plasma membrane proteins using Fur4 as a model substrate. Collectively, our data suggest that Ubp2 is not simply a negative regulator of Rsp5, but rather is engaged in a more complex and active regulatory relationship with Rsp5 at the MVB.

## Results

### Ubp2 Physically Interacts with Rsp5 and Rup1

**Figure 1:**
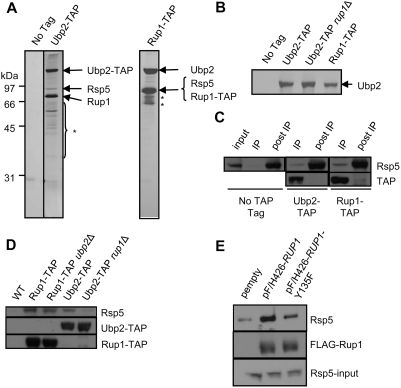
Ubp2, Rsp5, and Rup1 interact physically. (A) Silver-stained SDS polyacrylamide gel showing affinity purified Ubp2-TAP and Rup1-TAP protein complexes. Arrows indicate polypeptides identified by gel band excision followed by MALDI-ToF mass spectrometry. The asterisk indicates degradation products of Ubp2 and Rup1. (B) Ubp2 recovery from Ubp2-TAP, Ubp2-TAP *rup1Δ*, and Rup1-TAP strains. Arrow indicates gel band identified as Ubp2 by MALDI-ToF mass spectrometry. (C) Co-immunoprecipitation of a fraction of cellular Rsp5 with Ubp2 and Rup1. Cell extracts were depleted of endogenously TAP-tagged Ubp2 or Rup1 with IgG (IP lanes), and the remaining soluble Rsp5 (post-IP lanes) and bait proteins probed by Western blot with anti-Rsp5 antibodies which also recognized the protein A portion of the TAP tag. The ‘input’ lane shows the Rsp5 level in the extract prior to IP. (D) Rup1 tethers Ubp2 to Rsp5. IgG-based immunoprecipitation of Rup1-TAP and Ubp2-TAP from WT, *ubp2*Δ or *rup1*Δ strains. The co-purification of Rsp5 with the baits was determined by Western blotting using anti-Rsp5 antibodies. (E) Plasmids expressing FLAG(F/H)-tagged wildtype Rup1 or the Y135F point mutant were transformed into cells lacking endogenous Rup1. After immunoprecipitation with anti-FLAG antibodies, Rup1 and Rsp5 were detected by Western blotting. The bottom panel show the expression levels of Rsp5 in whole cell extracts by Western blotting against Rsp5.

Neither Ubp2 or Rup1 are essential for normal cell growth and deletion of either gene does not result in any obvious growth defects or overt morphological phenotypes [15], [25]. Hence, to investigate the possible role(s) of Ubp2 in yeast, we performed large-scale tandem affinity purifications (TAP) to isolate endogenous Ubp2 from *S. cerevisiae* strains bearing a C-terminal TAP tag [26]. Stably interacting partner proteins were resolved by SDS-PAGE, stained with silver, and identified by peptide mass fingerprinting using MALDI-TOF (matrix-assisted laser desorption/ionization-time of flight) mass spectrometry. Two polypeptides, encoding the E3 ubiquitin ligase Rsp5, and a protein of previously unknown function, YOR138c, which we have named Rup1 (Rsp5-Ubp2 interacting Protein 1), were found to reproducibly and specifically co-purify with Ubp2 (Figure 1A). Neither of these binding partners were detected in parallel purifications from an untagged negative control strain or from several hundred other TAP-tagged bait proteins [27]. These data were independently confirmed by highly-sensitive gel-free tandem mass spectrometry-based peptide shotgun sequencing (LC-MS/MS) of an aliquot of each protein mixture after in solution digestion with trypsin (http://tap.med.utoronto.ca/). Conversely, no known substrate or other previously reported Rsp5-interacting proteins [28]–[31] co-purified reproducibly with either Ubp2-TAP or Rup1-TAP (data not shown).

To further validate these results, we likewise performed reciprocal affinity purification using a strain expressing endogenous TAP-tagged Rup1 (a viable C-terminal Rsp5-TAP strain could not be generated). As expected, Rup1-TAP co-purified with a seemingly identical amount of Ubp2 and with Rsp5, albeit in an apparently sub-stoichiometric level (Figure 1A). Since immunoprecipitation of either Ubp2-TAP or Rup1-TAP resulted in a near-identical yield of Ubp2 (Figure 1B), the interaction between Ubp2 and Rup1 is seemingly complete and likely equimolar. In contrast, a substantial fraction of Rsp5 remained in solution and was not recovered with either Ubp2-TAP or Rup1-TAP after depletion of TAP-tagged proteins with IgG beads from soluble cell extracts (Figure 1C), indicating that a substantive fraction of Rsp5 exists in alternate protein complexes, or that Rsp5 binding is transient or labile. The relative proportion of cellular Rsp5 pool present in a complex with Ubp2 and Rup1 is estimated to be ∼10% of total. Since we have shown that only a modest fraction of the total cellular pool of Rsp5 is stably bound to Ubp2/Rup1, Ubp2, and by extension Rup1, may be involved in only a subset of Rsp5-dependent pathways.

To further investigate the structure-function dependencies of the interaction of Ubp2/Rup1 with Rsp5, we constructed TAP-tagged strains bearing a targeted genomic deletion in either Ubp2 or Rup1. The composition of the resulting affinity-isolated protein complexes were then examined by Western blotting. Whereas Rsp5 remained bound to Rup1-TAP in the absence of Ubp2 (Figure 1D), Ubp2-TAP did not detectably precipitate Rsp5 in the absence of Rup1. These results support a previous report [23] indicating that Rup1 likely mediates, at least in part, the interaction of Ubp2 to Rsp5. Consistent with this, we identified a putative WW domain consensus binding motif, PPPSY, encoded by amino acids 131–135 of Rup1. We generated a site-specific point mutant in which the invariant core tyrosine residue 135 was substituted with a phenylalanine (Y135F), and expressed this from an episomal plasmid in a strain lacking endogenous *RUP1*. This type of mutation (Y→F) has previously been shown to markedly reduce or even abolish binding of other WW domain proteins to their interacting partners bearing analogous PY motifs [32], [33]. Indeed, as expected, the interaction of this mutant variant with Rsp5 was impaired, although not abolished, relative to the wild-type control (Figure 1E), implying that the motif mediates in part the recruitment of Rsp5. Similar results were obtained after expression of the Rup1 Y135F mutant in a *ubp2Δ* deletion mutant background (data not shown), indicating the residual binding was not mediated by Ubp2.

### UBP2 and RUP1 Interact Genetically with RSP5

**Figure 2:**
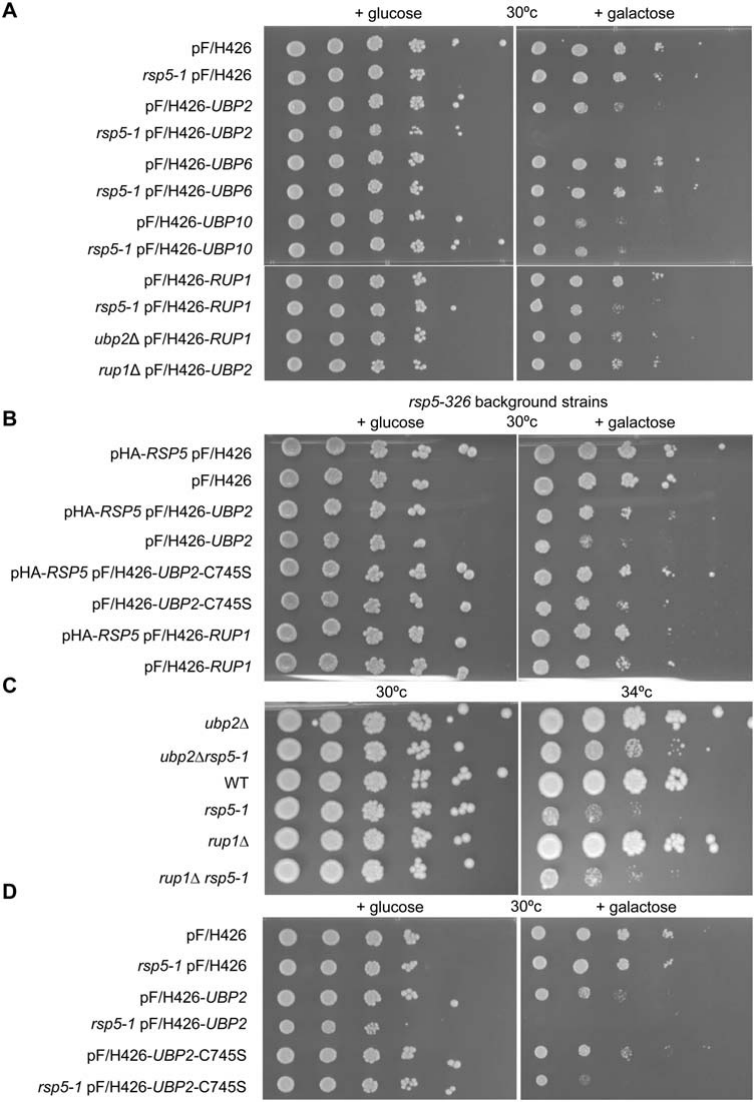
UBP2 and *RUP1* interact genetically with *RSP5*. (A, B, D) The indicated over-expression plasmids, driven by a galactose inducible promoter, were transformed into either wildtype or mutant strains. Cell cultures were then serially diluted 10-fold (starting OD_600_ of 0.5) and spotted onto SC-URA media containing glucose (non-inducing) or galactose (inducing). (B) Growth of *rsp5-326* mutant cells bearing pHA-*RSP5* (a low copy, but not galactose responsive plasmid), and/or a second overexpression plasmid as indicated. (C) Growth of cells deleted for either *UBP2* and *RUP1* alone, or in combination with an *RSP5* temperature sensitive allele (*rsp5-1*), on rich media (YPD) at either 30°c (permissive for *rsp5-1*) or 34°c (semi-permissive for *rsp5-1*).

To further explore the relationship of Ubp2 and Rup1 to Rsp5-related functions, we examined the effects of inducible ectopic overexpression of either Ubp2 or Rup1 in a strain harbouring a temperature-sensitive hypomorphic allele of *RSP5* (*rsp5-1*) [34], as well as in a wild-type strain, looking for enhancement or suppression of the conditional slow-growth phenotype. As seen in the limiting dilution series shown in Figure 2A, Ubp2 overexpression was moderately toxic to the control strain as compared to an empty control vector, while the *rsp5-1* mutant was found to be extremely hypersensitive. This synthetic dosage lethality was specific to *UBP2*, as the *rsp5-1* strain did not shown any hypersensitivity to overexpression of two other unrelated deubiquitinating enzymes, *UBP6* and *UBP10* (Figure 2A). Indeed, although overproduction of *UBP10* caused a noticeable growth defect in wildtype cells, this defect did not worsen when combined with the *rsp5-1* mutation, in striking contrast to *UBP2*. While wildtype yeast were not detectably sensitive to the overexpression of Rup1, *rsp5-1* and *ubp2Δ* mutants displayed modest hypersensitivity. On the other hand, a deletion of *RUP1* resulted in a reduction in the growth impairment caused by the overexpression of *UBP2*. These data support the notion that Rup1 functions coordinately with, but not equivalently to Ubp2. To confirm that these genetic interactions were not specific to the *rsp5-1* allele, another allele, *rsp5-326* [35] was tested (Figure 2B). All of the above interactions were similar in this mutant background. When Rsp5 was expressed from a low copy number plasmid, pHA-*RSP5* [36], in *rsp5-326* strains, the growth phenotypes seen were rescued to wildtype levels, indicating that these phenotypes were specific to *RSP5*.

Consistent with this apparent functional antagonism (Figure 2A; [23]), deletion of the *UBP2* open reading frame partially rescued the temperature-sensitivity of an *rsp5-1* mutant strain (Figure 2C), although deletion of *RUP1* had no observable affect. Taken together, these results are consistent with a recently proposed model [23] wherein Ubp2 directly antagonizes Rsp5 activity, and imply that Rup1 likely serves as a less critical ancillary role such as facilitating the interaction of Ubp2 with Rsp5 or possible substrates.

### Ubp2 Catalytic Activity is Important, but Not Essential, for Synthetic Dosage Lethality

A key characteristic of Ubps is the presence of an evolutionarily conserved catalytic domain [15]. To test whether the catalytic activity of Ubp2 was necessary for the antagonistic interaction observed with Rsp5-deficient strains, we mutated cysteine 745, a critical residue of the core Cys box motif conserved across all members of the Ubp family, to serine, which is predicted to result in a severe or complete loss of deubiquitinating capacity [19], [37]. As expected, we observed that overexpression of this putatively inactive mutant, *ubp2* C745S, led to a marked reduction in the slow-growth phenotype caused by overexpression of *UBP2* in either *rsp5* strain background (Figure 2, B and D), suggesting that inappropriate or excessive deubiquitination activity is largely, but not exclusively, responsible for the functional antagonism observed.

### ubp2Δ Cells are Sensitive to 5-Fluorouracil (5-FU)

**Figure 3:**
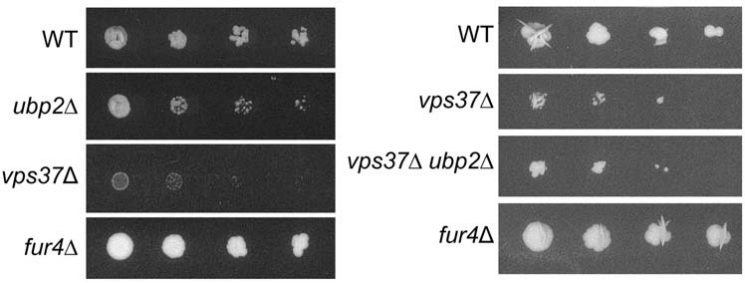
Loss of *UBP2* results in hypersensitivity to 5-fluorouracil (5-FU). Wildtype, *ubp2*Δ, *vps37*Δ, *fur4*Δ single mutants, and *vps37*Δ*ubp2*Δ double mutant cells were transformed with a *URA3* plasmid and subsequently tested for sensitivity to 5-FU. Exponentially growing cells were serially diluted 10-fold (starting OD_600_ ∼0.7) and spotted onto SD plates with 5 µM 5-FU. Plates were imaged either after 4 days (*left panel*) or 7 days (*right panel*).

Given that Rsp5 has a well-established role in the internalization, trafficking, and subsequent vacuolar degradation of membrane bound transporters [5], we explored the possibility that Ubp2 might also modulate the efficiency or dynamics of these ubiquitination-dependent events. One well characterized substrate of Rsp5 is the uracil permease Fur4. *rsp5* mutants have increased plasma membrane steady state amounts of Fur4. The same is true for mutants in class E genes that accumulate an abnormal compartment, the class E compartment, from which Fur4 recycles to plasma membrane. All these mutants display increased sensitivity to the drug 5-Fluorouracil (5-FU) [12], which is imported by Fur4. 5-FU is a toxic uracil analogue which inhibits the nucleotide synthetic enzyme thymidylate synthase and incorporates aberrantly into cellular RNA and DNA [38], leading to cell death. We checked *ubp2*Δ for potential sensitivity to 5-FU, using a class E mutant, *vps37*Δ, as a control (Figure 3). As expected, cells in which the transporter is absent (*fur4*Δ) are completely resistant to the drug (Figure 3; [39]). Compared to wildtype cells, *ubp2*Δ mutants are partially sensitive to the drug. However, they were not as sensitive as cells with a deletion in *vps37*Δ. The sensitivity of *vps37*Δ cells is not affected when combined with a mutation in *UBP2*, suggesting that *VPS37* and *UBP2* may act at the same level. These data show that Fur4 may be stabilized at the plasma membrane of *ubp2*Δ cells, leading to an increased sensitivity to 5-FU. The stabilization may not be complete as the sensitivity of *ubp2*Δ is less than that of strains defective in various sorting steps along the endocytic pathway.

### ubp2Δ Cells Have Increased Steady State Amounts of Plasma Membrane Localized Fur4

**Figure 4:**
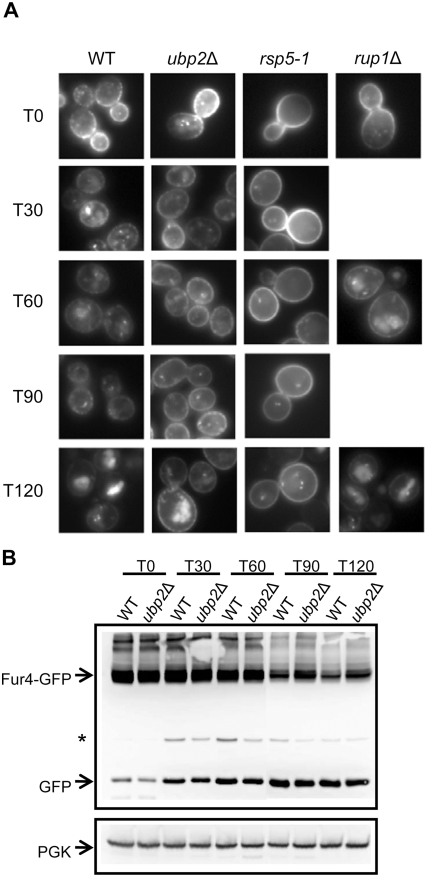
Uracil-induced Fur4 sorting is perturbed in *ubp2Δ* mutant cells. A *FUR4*-GFP fusion reporter was transformed into cells, which were then grown in raffinose overnight. Galactose was added (OD_600_ = 0.6) for 2 hours to induce synthesis, followed by glucose for 10 min to chase Fur4-GFP to the plasma membrane, and then uracil (40 µg/ml) to trigger transporter internalization. (A) Fur4-GFP signal viewed by fluorescence microscopy at the indicated time points (min) after uracil addition. T0 indicates cells immediately before addition. (B) Fur4-GFP protein levels as measured by Western blotting. Total protein extracts were generated from cells harvested at the indicated time points following uracil addition. Fur4-GFP was probed with anti-GFP antibodies. 3-phosphoglycerate kinase (PGK) was used as a loading control. The asterisk indicates an unknown background band.

In order to define whether the 5-FU sensitivity of *ubp2*Δ cells indeed comes from defects in trafficking, or merely from impaired RNA metabolism [40], we followed the fate of a GFP-tagged version of Fur4 in *ubp2*Δ and *rup1*Δ cells, using as control *rsp5* mutant cells (Figure 4, A and B). A galactose-inducible version of Fur4-GFP was similarly targeted to the plasma membrane after galactose induction in all the strains. Glucose was then added to stop Fur4-GFP synthesis and chase to the plasma membrane any Fur4-GFP still in the secretory pathway. Uracil was then added to trigger Fur4-GFP endocytosis. Cells were harvested at various time points after the addition of uracil, and subjected to whole-cell imaging (Figure 4A). Total protein extracts were also prepared in parallel for Western blotting against Fur4-GFP in the case of wildtype and *ubp2*Δ cells (Figure 4B). Uracil triggered a progressive loss of plasma membrane GFP fluorescence in wildtype cells, a transient apparition of punctuate intracellular fluorescent dots (likely endosomes), followed by apparition of luminal vacuolar fluorescence (corresponding to free GFP, not immediately degraded by vacuolar proteases). Fur4-GFP displayed almost the same fate in *rup1*Δ cells. In contrast, in *rsp5-1* cells, even at a permissive temperature, plasma membrane fluorescence was still detectable after two hours of uracil treatment, together with very faint vacuolar fluorescence. In *ubp2*Δ cells, the situation was intermediate between that observed in wildtype and *rsp5* cells. Plasma membrane staining was still observed after 90 min of uracil treatment, and after two hours, both plasma membrane and vacuolar fluorescence were evidenced. In agreement with this observation, Fur4-GFP degradation was delayed in *ubp2*Δ compared to wildtype cells (Figure 4B).

**Figure 5:**
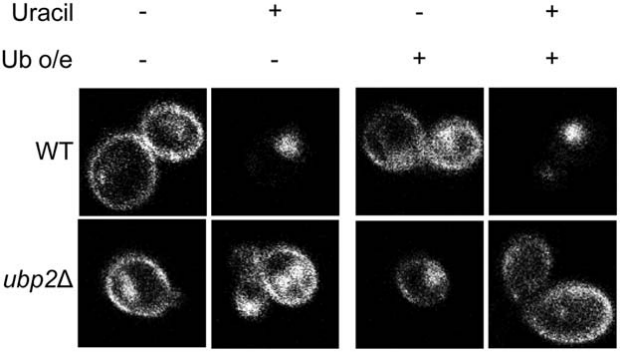
The overexpression of ubiquitin does not rescue the sorting defect in *ubp2*Δ cells. p*FUR4*-GFP and a plasmid overexpressing ubiquitin (+) or an empty plasmid (−) were transformed into cells. Strains were grown in sucrose, and diluted to OD_600_ = 0.5 in media containing galactose and 0.1 mM copper sulphate (to overexpress ubiquitin) and grown for 4 hours. Fur4 transcription was stopped by adding glucose for 1 hour to chase Fur4 to the plasma membrane. Uracil (40 µg/ml) was then added to induce Fur4 internalization. The GFP signal was viewed by fluorescence confocal microscopy before (−) and at 60 min after (+) uracil addition. Note that the left panel contains the same images as Figure 7, as the experiments were done in parallel.

Deubiquitinating enzymes such as Ubp4 (Doa4) have previously been linked to the overall recycling of ubiquitin prior to the proteasomal or vacuolar degradation of substrates [18], [41]. Depletion of the free ubiquitin pools in a yeast cell has the potential to lead to defects in protein sorting from the plasma membrane [6], presumably by indirectly reducing the efficiency of Rsp5-mediated ubiquitination at various steps of the sorting pathway. To exclude the possibility that the Fur4-GFP sorting defect observed in *ubp2*Δ mutants was an artifact due to a general reduction in free ubiquitin levels, we assessed whether the phenotype could be suppressed by the forced overexpression of ubiquitin from a plasmid using a strongly inducible copper promoter. Impaired sorting in the *ubp2* deletion mutant was not restored (Figure 5). This data, along with other previously reported data showing that *ubp2* mutants exhibit wildtype levels of free ubiquitin [17], indicate that the impaired kinetics of Fur4-GFP sorting in *ubp2*Δ mutants are indeed likely a direct result of improper regulation of the sorting pathway, and not due to a deficit in cellular ubiquitin levels.

A deletion in *ubp2* could result in abnormal vesicle morphology leading to indirect defects in Fur4 sorting. To determine if this was the case, wildtype and *ubp2*Δ cells were labelled with the fluorescent lipid-binding dye FM4-64, washed, and endocytosis of the dye was examined. Compared to wildtype cells, *ubp2*Δ cells displayed a normal rate of sorting of the dye to endosomes and eventually to the vacuolar membrane (Figure S1). Normal endosome and vacuolar morphologies were also seen, although subtle differences may not be detected with this assay. Taken together, these data implicate Ubp2 in the intracellular sorting of Fur4, specifically at a step in the trafficking of Fur4-GFP from the plasma membrane to the vacuole.

### ubp2Δ Cells Display Normal Plasma Membrane Fur4 Ubiquitination and Internalization

Plasma membrane stabilization of Fur4 subsequent to conditions normally triggering its endocytosis can be observed in two types of situations: in mutants impaired in the internalization step of endocytosis [10], [13], and in class E mutants with impaired MVB sorting. In the latter case, Fur4 recycles from the endosomal class E compartment back to plasma membrane. In order to decipher the origin of plasma membrane stabilization of Fur4-GFP in *ubp2*Δ cells, we first searched, using *vps* class E mutants, for conditions preventing any recycling.

Fur4-GFP was expressed in wildtype and *vps37*Δ cells, and cells were subjected to carbon starvation (CS) to induce internalization of Fur4-GFP from the plasma membrane. Glucose (CA), was then added, to induce recycling of Fur4 back up to the plasma membrane, in the presence or absence of an inhibitor of protein synthesis, cycloheximide (CHX). Fur4-GFP localization was followed by both fluorescence microscopy (Figure S2A) and by monitoring uracil uptake (Figure S2B). In both WT and *vps37*Δ cells, Fur4-GFP was internalized completely after carbon starvation as expected (Figure S2A, [12]). Fur4-GFP was rapidly targeted back up to the plasma membrane in *vps37*Δ cells after the addition of glucose but not upon addition of glucose+CHX. Recycling only occurred in *vps37*Δ cells, as the mutation blocked the entry of Fur4-GFP into the vacuolar lumen; in wildtype cells all Fur4-GFP was targeted to vacuolar lumen, where it was degraded [12]. As expected, uracil uptake increased over 60 minutes post-glucose addition in *vps37*Δ cells in the absence of CHX (Figure S2B). Taken together, these data show that recycling of Fur4-GFP cannot be detected in the presence of CHX, although the exact mechanism is unknown. Thus, triggering endocytosis of Fur4-GFP in the presence of CHX was a good condition in which to define the level of action of Ubp2.

**Figure 6:**
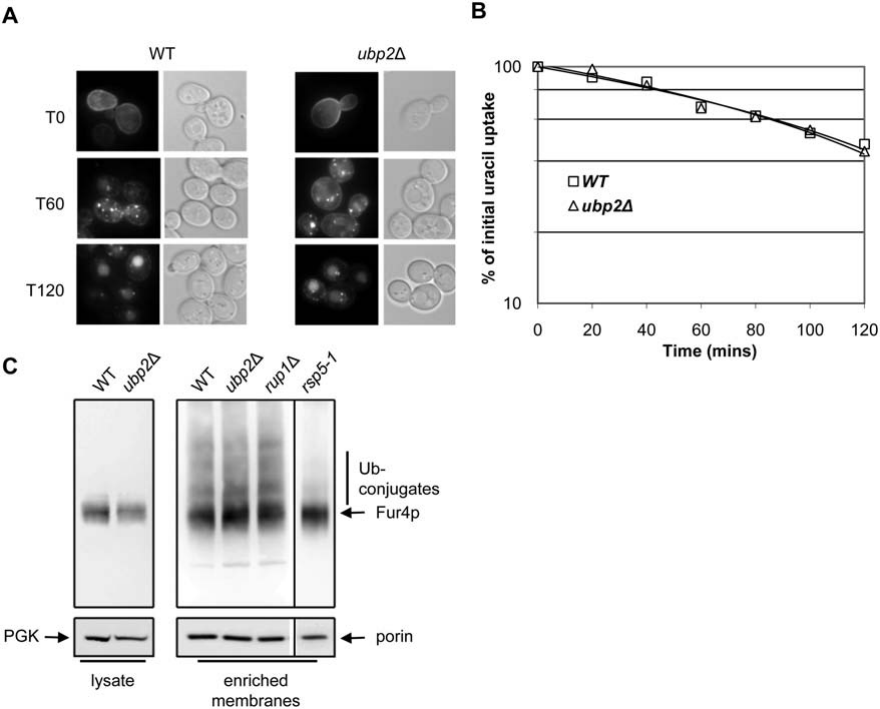
*UBP2* is not necessary for cycloheximide (CHX) triggered internalization of Fur4. (A) p*FUR4*-GFP bearing cells were grown in raffinose overnight. Galactose was added for 2 hours to induce synthesis, and glucose was then added for 10 min to chase Fur4-GFP to the plasma membrane. CHX (0.1mg/ml) was added and GFP signal examined by fluorescence microscopy and Nomarski optics. Time refers to the time after addition of CHX, with 0 min as pre-induction. (B) Uracil uptake was measured at different times after the addition of CHX in WT and *ubp2*Δ strains. Results are expressed as a percentage of the initial uracil uptake, and plotted on a log scale. (C) Fur4 ubiquitin profile at the plasma membrane is unchanged in *ubp2Δ* mutants. p*FUR4* was transformed into WT, *ubp2*Δ, *rup1*Δ, and *rsp5-1*, and cells, which were grown in raffinose overnight. Expression from p*FUR4* was induced for 90 min with galactose before adding glucose for 15min to chase Fur4 to the plasma membrane. Total protein extracts (lysate) and enriched membrane fractions were collected and analyzed by Western blotting to visualize Fur4 and Fur4-ubiquitin conjugates. 3-phosphoglycerate kinase (PGK) and porin, a mitochondrial membrane protein, were used as loading controls.

After two hours of galactose induction of Fur4-GFP followed by glucose, CHX was added to wildtype and *ubp2*Δ cells. The fate of Fur4-GFP was then followed by fluorescence imaging and uracil uptake measurement (Figure 6, A and B). Plasma membrane fluorescence disappeared in a similar way in wildtype and *ubp2*Δ cells (Figure 6A), and the loss of uracil uptake occurred with identical rate in both type of cells (Figure 6B). Endocytic internalization of Fur4 is thus normal in *ubp2*Δ cells. In agreement with the observation of a normal rate of internalization, the pattern of ubiquitination (regularly spaced Fur4 species evidenced above the main Fur4-GFP signal) on Western blots of plasma membrane enriched fractions was identical in wildtype and *ubp2*Δ cells (Figure 6C). This is in comparison to *rsp5-1* cells, which showed a distinct lack of Fur4-ubiquitin conjugates as expected. This data implies that Ubp2 is likely not involved in the maintenance or deubiquitination of Fur4 at the plasma membrane.

### Ubp2 is Involved in Efficient Fur4-GFP Ubiquitination in the Late Steps of Endocytosis

If Ubp2 is not required for plasma membrane ubiquitination and internalization, it must therefore be required for subsequent steps of trafficking. In a survey of all membrane bound Fur4 in the cell, the ubiquitin status of Fur4 appears to be unaffected in *ubp2*Δ cells (data not shown). As any subtle and rapid ubiquitination/deubiquitination events may have been missed, and in order to more closely decipher the origin of the Fur4 trafficking defect in *ubp2*Δ cells, we investigated the role of ubiquitination in uracil-induced endocytosis.

**Figure 7:**
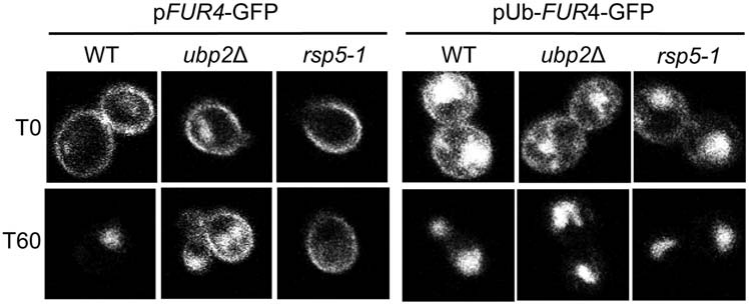
Ub-Fur4-GFP is correctly sorted at the MVB in *ubp2*Δ mutant cells. p*FUR4*-GFP or pUb-*FUR4*-GFP expression plasmids were transformed into WT and *ubp2*Δ cells. Strains were grown in sucrose, diluted (OD_600_ = 0.5) in media containing galactose and Fur4 synthesis induced for 4 hours. Transcription was stopped by adding glucose for 1 hour to chase Fur4 fusion reporters to the plasma membrane. Uracil (40 µg/ml) was then added to trigger internalization. GFP signal was viewed by fluorescence confocal microscopy both before (T0) and 60 min after uracil addition. (Note: cells in the left panel were also transformed with a control vector (YEp46Δ) and incubated with 0.1 mM copper sulphate, as the experiment was done in parallel to that shown in Figure 5.)

Defects in Rsp5-dependent, ubiquitin-dependent trafficking of a number of membrane-bound proteins, either at plasma membrane, or at MVB, can be bypassed using an in frame fusion of ubiquitin to these various cargoes. This is notably the case for Fur4 [13]. Hence, we examined whether expression of the Fur4-GFP reporter construct bearing ubiquitin as an N-terminal fusion (pUb-*FUR4*-GFP) could likewise suppress the sorting defect following the loss of Ubp2 activity. Control experiments (Figure 7) confirmed that, as expected, the fusion construct rescued, at least partially, the internalization defect seen in *rsp5-1* cells at 60 minutes post-uracil addition. Likewise, the Ub-Fur4-GFP fusion protein was also efficiently cleared from the plasma membrane in the *ubp2* deletion mutants, as the fluorescence signal detected at the plasma membrane was virtually absent one hour after uracil induction. The concomitant increase in the background signal intensity detected in the vacuoles of *ubp2*Δ cells was expected due to missorting of nascent Ub-Fur4-GFP to the vacuole directly from the Golgi [13]. Taken together, these data suggest, albeit somewhat paradoxically, that the loss of Ubp2 activity impairs proper Rsp5-mediated ubiquitination of Fur4-GFP, likely at the level of MVB sorting. This sorting step was restored with Ub-Fur4-GFP, and the protein, correctly sorted to MVB, did not recycle to the plasma membrane.

### Ubp2 May Modulate the Sorting of Ste2

**Figure 8:**
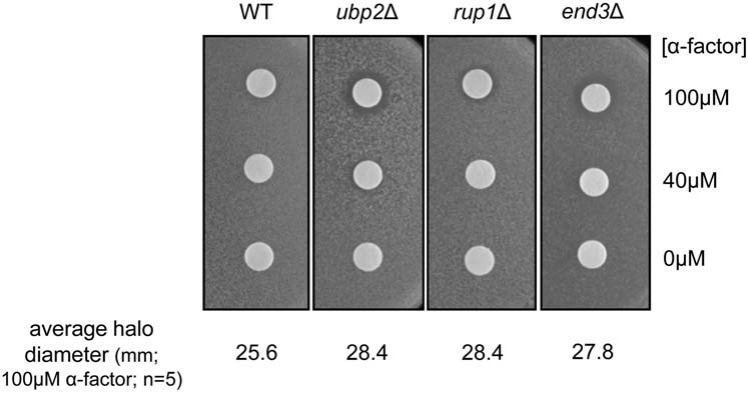
*ubp2*Δ cells are sensitive to alpha-factor consistent with a defect in Ste2 receptor sorting. α-factor (5 ul of 100 µM, 40 µM or water alone) was spotted onto sterile filter disks, and placed onto solid media containing WT, *ubp2*Δ, *rup1*Δ, or *end3*Δ *MATa* cells. Plates were then incubated at 30°C for one day and imaged. Halo diameters (mm) were measured and averaged from five replicates of 100 µM alpha factor spots.

The yeast α-factor receptor, Ste2, is another well-characterized substrate of Rsp5 [42]. Similar to Fur4, Ste2 is ubiquitinated, internalized, and subsequently degraded in the vacuole upon substrate-binding [42]–[44]. Defects in the sorting and degradation of Ste2 result in hypersensitivity to α-factor [45], as the presence of α-factor triggers cell cycle arrest in *MATa* recipient cells [46]. When a disk of α-factor is spotted on a lawn of *MATa* cells, a gradient zone of growth inhibition forms, termed a halo. *ubp2* deletion mutants exhibit hypersensitivity to α-factor by halo assay as compared to a WT control strain (Figure 8), suggesting a defect in clearing Ste2 from the plasma membrane of these cells. Surprisingly, the sensitivity of *ubp2*Δ cells to α-factor was slightly greater than in *end3* deletion mutants lacking an endocytic machinery component, which was previously found to be sensitive to α-factor [45], and *rup1*Δ cells have a sensitivity similar to *ubp2*Δ cells. These data suggest that Ubp2 modulates the ubiquitination and sorting of other plasma membrane associated proteins other than Fur4, but this effect may happen with slightly different kinetics depending on the cargo. The possible involvement of Rup1 in Ste2 transport is currently unclear and will be subject to further study.

## Discussion

Despite the rapidly expanding interest in DUBs as likely critical regulators of core cellular processes such as protein trafficking [47], [48], the physiological roles and targets of most of the known or predicted deubiquitinating enzymes remain unknown. In this report, we sought to confirm and extend the observations of previous studies regarding the molecular associations and putative functions of Ubp2 [23], [24], [49]. In principle, most of the genetic and physical interaction data reported in this study and previously by another group [23] point to an antagonistic, and presumably regulatory relationship with Ubp2/Rup1 counteracting Rsp5. In light of this perspective, the finding that *ubp2*Δ mutants have a defect in Rsp5-mediated transporter and receptor sorting reported here is especially surprising.

The accumulation of Fur4-GFP seen at the plasma membrane under uracil induced conditions could be due to a defect in internalization or an increase in recycling of Fur4 to the plasma membrane. We see that the internalization step itself does not seem to be impaired. Since the defect in Fur4-GFP trafficking appears only in recycling permissive conditions in *ubp2*Δ cells, our data imply that the accumulation of Fur4-GFP at the plasma membrane is most likely due to a more efficient recycling of the permease. An aberrant accumulation of membrane proteins at the plasma membrane can result from a defect in MVB formation, as has been reported with ESCRT mutants [12]. Therefore, it is likely that *ubp2*Δ mutants have a defect in internalization into MVBs. This defect is not complete, however, as Fur4-GFP eventually reaches the vacuolar lumen following a temporal lag (i.e. after 120 minutes post uracil addition versus 30 minutes in WT cells). Further evidence that places Ubp2 at the MVB sorting step is the fact that sensitivity to 5-FU does not seem to worsen in a *vps37*Δ*ubp2*Δ double mutant when compared to a *vps37*Δ single mutant alone. In addition, Ubp2, Rup1, and Rsp5 have been shown in other studies to be present at the MVB. Rsp5 was found to be located at various sites within the endocytic pathway, including plasma membrane invaginations, late endosomes, and MVBs by colocalization with Pep12, an endosomal marker, and Vps32, a component of an ESCRT complex located at the MVB [50]. Rsp5, Rup1 and Ubp2 have also been shown to interact with Hse1 [49], a component of the ESCRT-0 complex located at the MVB. In addition to Fur4, we see a role for Ubp2 in the proper clearance of another membrane-bound protein, Ste2, from the plasma membrane, suggesting Ubp2 is generally required for efficient sorting.

Consistent with these results, Ubp2 has recently been shown to be important for the Rsp5-mediated trafficking of a vacuolar protease, carboxypeptidase S (Cps), which is sorted to the vacuolar lumen from the Golgi via the MVB pathway [49]. In addition, a role for Ubp2 in properly sorting the membrane bound amino acid permease Gap1 directly from the Golgi to the MVB has also been reported [49]. In this study, we establish, at least in the case of Fur4 (and possibly for Ste2), the additional involvement of Ubp2 in sorting of endocytic cargo from the plasma membrane to the MVB. It seems, therefore, that Ubp2 has a role in modulating Rsp5-mediated trafficking of various types of membrane associated cargo.

What then, specifically, is the role of Ubp2? Although Ubp2 has been shown to deubiquitinate a non-essential ER-membrane bound transcription factor, Spt23, *in vitro* [23], there is as yet no evidence for the physiological processing of Spt23 *in vivo* (M.L.; unpublished observations). Indeed, counterintuitive to expectation, our observation that a ub-Fur4 fusion can rescue the sorting defect in *ubp2*Δ mutants suggests inefficient or improper ubiquitination of the internalized transporter.

One possibility is that Ubp2 may be involved in modulating the efficiency of Rsp5 ubiquitination, by deubiquitinating auto-ubiquitinated Rsp5, or by sequestering Rsp5 to a specific cellular compartment or substrate. Deubiquitination of Rsp5 would lead to the subsequent stabilization of the E3 ub-ligase as is the case for many previously identified E3-DUB physically interacting pairs [51], [52]. However, this appears unlikely, as no detectable decrease in Rsp5 levels was detected in a *ubp2*Δ mutants (M.L.; unpublished observations), and Rsp5 has not been reported to be (auto)ubiquitinated *in vivo*.

A second possibility is that Ubp2 may be involved in ubiquitin chain length editing of internalized receptors and transporters at the MVB. Most substrates of the endocytic pathway are covalently attached with single ubiquitin moieties or short K63-linked ubiquitin chains [6], [53]–[55], although a Vps pathway substrate with a K63-linked polyubiquitin chain has been recently reported [56]. The recent finding that Ubp2 can efficiently and preferentially deubiquitinate K63 linked chains [24] suggests a possible role for Ubp2 in editing Rsp5-catalyzed polyubiquitin chains down to a length suitable for recognition by various proteins in the endocytic/ESCRT machinery. Although a difference in the ubiquitin profile of Fur4 in a WT versus *ubp2*Δ mutants was not detected, our Western blot assay represented the sum of all membrane compartments in the cell, and may not be sensitive enough to visualize a subtle kinetic defect at the MVB. It would be interesting and informative to look at the ubiquitination of Fur4 in conditions where more subtle differences in ubiquitination levels at the MVB could be detected.

A third, and more likely (although currently speculative) possibility is a role of Ubp2 in the deubiquitination of the cargo (e.g. Fur4) right at the MVB, which is then followed by a second, subsequent re-ubiquitination by Rsp5 for efficient sorting into the MVB. In support of this model, it was previously reported that Rsp5-dependent ubiquitination of cargo must occur at the MVB stage for proper sorting into MVBs [57]. Further support for this model is our data showing a physical interaction between Rsp5 and Ubp2 through tethering by Rup1, allowing for a close physical proximity between the Ub ligase and the DUB at the MVB. Another DUB, Doa4, also appears to play a somewhat related, but different, role at the MVB, as deubiquitination by Doa4 itself is not sufficient for the proper re-ubiquitination of Fur4. Although it has not yet been shown conclusively that deubiquitination must occur prior to cargo re-ubiquitination, this will be an interesting area to address in the future. In addition to cargo deubiquitination, we cannot currently rule out an (additional) indirect role of Ubp2 on the sorting machinery itself, adding another possible level of complexity in the regulation of trafficking. We are also unable to conclusively exclude a role for Ubp2 at the plasma membrane, as there might have been subtle defects in *ubp2*Δ cells at the internalization step of endocytosis that remained undetected in our experimental conditions.

The role of Rup1 also remains enigmatic. Residual Ubp2-Rsp5 binding was detected in the absence of Rup1, implying that Ubp2 and Rsp5 can interact, either directly, or possibly through another unknown adaptor protein. This could explain why Rup1 is not required for proper MVB sorting, although a minor sorting defect not apparent in the assay conditions tested cannot be ruled out. Aspects of our genetic data imply that Rup1 has a positive role in Ubp2 activity. For example, deletion of *RUP1* suppressed, albeit only partially, the temperature-sensitive phenotype of *rsp5-1* mutants. The presence of a UBA domain in Rup1 is also suggestive. Ubiquitin-binding motifs, such as UBA or UIM (ubiquitin interacting motif) domains, are present in a sizeable fraction of proteins involved in intracellular protein trafficking [58], and have been suggested to facilitate the “passing-off” of ubiquitinated endocytic cargo or components from one functional module in the processing machinery to another [58], [59]. Hence, Rup1 could play a complex role at the MVB, in both recruiting ubiquitinated proteins to the ternary complex and tethering Ubp2 to Rsp5.

## Materials and Methods

### Strains and Growth Media

All transformations of yeast strains were done using a standard protocol [60], [61]. All yeast strains were grown in YP (yeast extract and peptone)+2% glucose and at 30°C unless otherwise specified. Relevant genotypes of strains are detailed in Table 1. TAP-tagged deletion mutant strains were constructed by direct transformation or by mating a relevant deletion strain with a suitable TAP tagged strain. Tetrads were pulled, and haploids containing both *HIS3* (TAP) and NAT-resistance (deletion) markers were selected. The presence of the deletion and the expression of the TAP-tagged protein were confirmed by PCR and Western blotting, respectively. *ubp2*Δ::*NatMX* and *rup1*Δ::*NatMX* strains were constructed as described in [62] by transformation with EcorRI-cut p4339 into cells or by PCR of the *NatMX* cassette from p4339 followed by transformation. For Figure 2, wildtype strains in 2A and 2D, and *rup1*Δ in 2C, are derived from FY56 which is isogenic to *rsp5-1.* For other figures, wildtype strains are BY4741. *ubp2*Δ*rsp5-1 and rup1*Δ*rsp5-1* were constructed either from direct transformation of the deletion cassette into *rsp5-1*, or by mating of haploids, tetrad dissection, and selection of haploids with the required genotype. *vps37*Δ was constructed as described in [63] and *vps37*Δ*ubp2*Δ was constructed by crossing single mutant strains.

**Table 1:** Yeast Strains Used in this Study.

Strain/Plasmid	Relevant Genotype	Origin
**WT**	BY4741 (*MATa his3*Δ*1 leu2*Δ*0 ura3*Δ*0 met15*Δ*0*)	[68]
**Ubp2-TAP**	*MATa his3Δ1 leu2Δ0 met15Δ0 ura3Δ0 UBP2::TAP-HIS3*	[69]
**Rup1-TAP**	*MATa his3Δ1 leu2Δ0 met15Δ0 ura3Δ0 RUP1::TAP-HIS3*	[69]
***ubp2*** **Δ**	*ubp2*Δ::*NATMX,* derived from Y3656 (*MATα can1Δ::MFA1pr-HIS3-MFα1pr-LEU2 his3Δ leu2Δ0 ura3Δ0 met15Δ0 lys2Δ0)*	This study, [70]
***ubp2*** **Δ**	*MATa his3*Δ*1 leu2*Δ*0 ura3*Δ*0 met15*Δ*0 ubp2*Δ::*KanMX3*	[25]
***rup1*** **Δ**	*rup1*Δ::*NATMX,* derived from Y3656 (*MATα can1Δ::MFA1pr-HIS3-MFα1pr-LEU2 his3Δ leu2Δ0 ura3Δ0 met15Δ0 lys2Δ0)*	This study, [70]
***rup1*** **Δ**	*MATa his3*Δ*1 leu2*Δ*0 ura3*Δ*0 met15*Δ*0 rup1*Δ::*KanMX3*	[25]
***rup1*** **Δ**	*rup1Δ::NATMX*, derived from FY56 (*MATα his4-912 ΔR5 lys2-128Δ ura3-52)*	This study, [71]
**Ubp2-TAP ** ***rup1*** **Δ**	*MATa UBP2::TAP-HIS3, rup1*Δ::*NATMX*	This study
**Rup1-TAP ** ***ubp2*** **Δ**	*MATa RUP1::TAP-HIS3, ubp2*Δ::*NATMX*	This study
***rsp5-1***	FW1808 (*MATα his4-912 ΔR5 lys2-128Δ ura3-52 rsp5-1*)	[71]
***rsp5-326***	*MATa leu2-3,112 ura3-52 his3-Δ200 trp1-Δ901 ade2-101 suc2-Δ9*	[35]
***ubp2*** **Δ ** ***rsp5-1***	*ubp2*Δ::*KanMX3 rsp5-1*	This study, [71]
***rup1*** **Δ ** ***rsp5-1***	*rup1*Δ::*KanMX3 rsp5-1*	This study, [71]
***fur4*** **Δ**	*MATa his3*Δ*1 leu2*Δ*0 ura3*Δ*0 met15*Δ*0 fur4*Δ::*KanMX3*	[25]
***end3*** **Δ**	*MATa his3*Δ*1 leu2*Δ*0 ura3*Δ*0 met15*Δ*0 end3*Δ::*KanMX3*	[25]
***vps37*** **Δ**	*vps37*::HIS	This study
***vps37*** **Δ** ***ubp2*** **Δ**	*vps37*::HIS *ubp2*Δ::*KanMX3*	This study

### Plasmids

Plasmids allowing for inducible overexpression of N-terminal affinity tagged proteins were constructed by recombineering essentially as previously described [64]. Briefly, full-length ORFs were amplified from yeast genomic DNA using primers similar to the Yeast Genepairs Primers (Research Genetics) bearing homology to the 5′ and 3′ ends of each ORF. The product from this first PCR reaction was re-amplified using primers which contain sequences homologous to those flanking a SmaI cloning site downstream of a galactose inducible promoter in a high copy yeast expression plasmid with a selectable *URA3* marker gene (pF/H426). The PCR product was integrated into the parental plasmid, linearized with SmaI, by co-transformation and *in vivo* homologous recombination, resulting in an in-frame fusion to a triple FLAG/His_10_/HA_2_ (F/H) epitope affinity tag immediately upstream of the ORF. Correct expression of the fusion protein was confirmed by Western blotting. The mutant constructs pF/H426-*UBP2*-C745S and pF/H426-*RUP1*-Y135F, bearing site specific point mutations were constructed using the QuikChange II Site-Directed Mutagenesis kit (Stratagene). Mutants were confirmed by dideoxy DNA sequencing. pHA-*RSP5* (p[HA-*RSP5, CEN, LEU2*]) was from [36]. The galactose inducible expression plasmids p*FUR4* (pFL38GalFUR4), p*FUR4*-GFP (pFL38GalFUR4-GFP), and ubiquitin-fusion construct pUb-*FUR4*-GFP (pFL38GalUb-*FUR4*-GFP; containing a variant ubiquitin with all its relevant lysine residues mutated to arginine to ensure that no additional ubiquitin can be added to the ubiquitin moiety, and glycine 76 replaced with a valine, to prevent proteolytic cleavage) were from [8], [13], [65]. The chromosome encoded uracil permease is produced in very small amounts and cells that expressed the permease under the Gal1 promoter from a centromeric plasmid were used for accurate measurement of the permease activity and for the immunodetection of the protein [7] The cooper-inducible ubiquitin expression plasmid pCUP1-Ub-TRP1 (YEp96) and the control vector (YEp46Δ) are from [66].

### TAP-tagged Protein Purification and Mass Spectrometry

Large-scale TAP-tagged protein purifications were separated on 10% polyacrylamide gels, and silver stained using a standard protocol. Subsequent analysis of the mixtures of purified proteins by both gel-free tandem mass spectrometry and gel-based MALDI-ToF mass spectrometry were performed essentially as previously described [27].

### Cell Lysis, MiniGel Electrophoresis and Western Blotting

Cells were harvested and lysed by glass bead beating in YEB (Yeast Extraction Buffer: 245 mM KCl, 1 mM EDTA, 5 mM EGTA, 50 mM Hepes-KOH pH 8.0, 10% glycerol, 2 mM DTT) containing an EDTA-free protease inhibitor cocktail (Complete; Roche) or YEB containing 350 mM KCl to decrease non-specific binding to antibody beads (Rup1 Y135F binding experiments) and centrifuged at 14 krpm at 4°C for 10 minutes to pellet insoluble debris. For the immunoprecipitation experiments, approximately 6 mg of total soluble protein extract (∼10 mg/ml) was then incubated for 1 hour at 4°C with anti-FLAG M2 Agarose affinity beads (Sigma-Aldrich) for F/H tagged strains, or with IgG Sepharose 6 Fast Flow beads (Amersham Pharmacia) for TAP-tagged strains. The beads were washed extensively, and tightly bound proteins were eluted with 15 ul of SDS sample buffer by heating at 90°C for 5 minutes. The samples were separated by electrophoresis on a 4–12% Bis-Tris polyacrylamide gel (Invitrogen), and the proteins transferred from the gel onto nitrocellulose membrane (Trans-Blot Transfer Medium; BioRad). The membranes were then extensively pre-blocked with a 5% milk protein solution, followed by overnight incubation with mouse monoclonal anti-HA antibody (12CA5 cell line; a kind gift from Mike Tyers, Samuel Lunenfeld Research Institute, Mount Sinai Hospital, Toronto, Ontario) to detect the TAP tag, rabbit anti-Rsp5 antibody (a kind gift from Linda Hicke [67]), or mouse anti-FLAG antibody (Sigma-Aldrich). Detection was performed by ECL (SuperSignal West Pico, Pierce) using suitable secondary antibodies conjugated to horseradish peroxidase. For the Fur4 immunoblots, whole cell lysates (Figure 6) were made by cell disruption followed by centrifugation at 3000 g, and total protein extracts (Figure 4B) were prepared by the NaOH-TCA lysis technique as previously described [10]. To better detect ubiquitinated species of Fur4, membrane enriched fractions of cells producing the lower molecular weight Fur4, and not the Fur4-GFP fusion were analyzed. Membrane-enriched protein fractions were prepared as previously described [18] except that cells were broken in a “One Shot” Cell Disrupter (Constant Systems LDT) at maximum pressure. Proteins in sample buffer were heated at 37°C, resolved by SDS-polyacrylamide gel electrophoresis in 10% acrylamide gels using tricine buffer, and transferred to nitrocellulose membranes. The membranes were probed with monoclonal anti-GFP antiserum (Roche), anti-PGK (Molecular Probes), rabbit anti-Fur4 antibody [10], or monoclonal antibodies against porin (Molecular Probes). Horseradish peroxidase-conjugated anti-mouse or anti-rabbit immunoglobulin G was used as the secondary antibody (Sigma) and was detected by enhanced chemiluminescence (ECL).

### Serial Dilutions

Strains were grown overnight to saturation in SC-URA or YP media containing 2% glucose, and diluted with sterile water to OD_600_ = 0.5. 10-fold dilutions with sterile water were then made, and 3 µl of the cell suspension spotted onto media plates containing rich (YP) or selective (SC-URA) media+2% glucose or 2% sucrose/1% galactose. Plates were incubated for 2–4 days at 30°C or 34°C and imaged. For 5-FU plates, cells were grown to OD_600_ ∼0.7. 10-fold dilutions were then made, and 20 µl of the cell suspension spotted onto media plates containing SD+5 µM 5-FU (Sigma). Plates were incubated for 4 or 7 days and imaged.

### Uracil uptake assay

Uracil uptake was measured as previously described [10], by incubating a 1 ml culture of exponentially growing cells with 5×10^−6^ M radiolabeled uracil for 20 s at 30°C. Cells were quickly filtered through Whatman GF/C filters, which were then washed twice with ice-cold water and counted for radioactivity. Fur4p activity was measured various times after the addition of cycloheximide (100 µg/ml; Sigma) as previously described [10].

### Microscopy

Cells grown to exponential growth phase in YNB medium was concentrated by a factor of ten by centrifugation. Cells were viewed immediately, without fixation, under a fluorescence microscope (type BY61, Olympus, Tokyo, Japan) and images captured with a digital camera. For Figures 5 and 7, fluorescence images of live yeast cells at a 512×512 pixel resolution were generated with a Leica DM IRBE confocal microscope using a 100× objective with immersion oil. Images were processed using Leica TCS software.

### α-factor Halo Assay

*MATa* cells from an overnight culture were added to pre-warmed (55°C) sterile 0.5% agar, vigorously mixed, and poured evenly as a thin layer onto plates containing solid YP+2% glucose media. After solidifying, sterile filter disks soaked with 5 ul of various concentrations of α-factor (Pepceuticals) were gently overlaid onto the cell lawn, followed by incubation at 30°C for one day prior to scoring.

Materials and Methods for Figures S1 and S2 are described in Supporting Information file Materials and Methods S1.

## Supporting Information

Figure S1 Endosome and Vacuolar morphology is normal in *ubp2*Δ cells. Cells were incubated with the lipid-binding fluorescent dye FM4-64 and washed. Endocytosis of the dye and endosome/vacuolar morphology were monitored by fluorescence microscopy and Nomarski optics at the indicated time points (min) after staining.
(1.96 MB TIF)

Figure S2 Recycling of Fur4-GFP cannot be detected in the presence of cycloheximide. WT and *vps37*Δ cells transformed with pFur4-GFP were cultured at 30°C and Fur4-GFP synthesis was induced for 90 min by adding galactose. Glucose was added to block Fur4-GFP synthesis (CC). 20 min later, cells were subjected to carbon starvation (CS) to trigger endocytosis of the permease for 60 minutes. Cultures were divided in two equal fractions. Glucose (CA) or glucose+cycloheximide (CA+CHX 0.1 mg/ml) was then added. (A) Cells were visualized by fluorescence microscopy at t = 0 (0'CC), after 60 minutes of carbon starvation (60'CS) and 60 minutes after the addition of carbon (60'CA) or carbon+cycloheximide (60'CA+CHX). Note that Fur4-GFP was efficiently internalized in all the strains upon CS. (B) Uracil uptake was measured 3, 10, 30 and 60 min after the addition of glucose. Results are expressed as a percentage of the initial uracil uptake measured immediately before carbon starvation, and plotted on a linear scale.
(0.46 MB TIF)

Materials and Methods S1 (0.04 MB DOC)

## References

[1] Hochstrasser M (1996). Ubiquitin-dependent protein degradation.. Annu Rev Genet.

[2] Sun ZW, Allis CD (2002). Ubiquitination of histone H2B regulates H3 methylation and gene silencing in yeast.. Nature.

[3] Hicke L (1997). Ubiquitin-dependent internalization and down-regulation of plasma membrane proteins.. Faseb J.

[4] Dupre S, Urban-Grimal D, Haguenauer-Tsapis R (2004). Ubiquitin and endocytic internalization in yeast and animal cells.. Biochim Biophys Acta.

[5] Horak J (2003). The role of ubiquitin in down-regulation and intracellular sorting of membrane proteins: insights from yeast.. Biochim Biophys Acta.

[6] Galan JM, Haguenauer-Tsapis R (1997). Ubiquitin lys63 is involved in ubiquitination of a yeast plasma membrane protein.. Embo J.

[7] Marchal C, Haguenauer-Tsapis R, Urban-Grimal D (1998). A PEST-like sequence mediates phosphorylation and efficient ubiquitination of yeast uracil permease.. Mol Cell Biol.

[8] Seron K, Blondel MO, Haguenauer-Tsapis R, Volland C (1999). Uracil-induced down-regulation of the yeast uracil permease.. J Bacteriol.

[9] Marchal C, Haguenauer-Tsapis R, Urban-Grimal D (2000). Casein kinase I-dependent phosphorylation within a PEST sequence and ubiquitination at nearby lysines signal endocytosis of yeast uracil permease.. J Biol Chem.

[10] Volland C, Urban-Grimal D, Geraud G, Haguenauer-Tsapis R (1994). Endocytosis and degradation of the yeast uracil permease under adverse conditions.. J Biol Chem.

[11] Katzmann DJ, Odorizzi G, Emr SD (2002). Receptor downregulation and multivesicular-body sorting.. Nat Rev Mol Cell Biol.

[12] Bugnicourt A, Froissard M, Sereti K, Ulrich HD, Haguenauer-Tsapis R (2004). Antagonistic roles of ESCRT and Vps class C/HOPS complexes in the recycling of yeast membrane proteins.. Mol Biol Cell.

[13] Blondel MO, Morvan J, Dupre S, Urban-Grimal D, Haguenauer-Tsapis R (2004). Direct sorting of the yeast uracil permease to the endosomal system is controlled by uracil binding and Rsp5p-dependent ubiquitylation.. Mol Biol Cell.

[14] Morvan J, Froissard M, Haguenauer-Tsapis R, Urban-Grimal D (2004). The ubiquitin ligase Rsp5p is required for modification and sorting of membrane proteins into multivesicular bodies.. Traffic.

[15] Baker RT, Tobias JW, Varshavsky A (1992). Ubiquitin-specific proteases of Saccharomyces cerevisiae. Cloning of UBP2 and UBP3, and functional analysis of the UBP gene family.. J Biol Chem.

[16] Wilkinson KD (2000). Ubiquitination and deubiquitination: targeting of proteins for degradation by the proteasome.. Semin Cell Dev Biol.

[17] Amerik AY, Li SJ, Hochstrasser M (2000). Analysis of the deubiquitinating enzymes of the yeast Saccharomyces cerevisiae.. Biol Chem.

[18] Dupre S, Haguenauer-Tsapis R (2001). Deubiquitination step in the endocytic pathway of yeast plasma membrane proteins: crucial role of Doa4p ubiquitin isopeptidase.. Mol Cell Biol.

[19] Cohen M, Stutz F, Belgareh N, Haguenauer-Tsapis R, Dargemont C (2003). Ubp3 requires a cofactor, Bre5, to specifically de-ubiquitinate the COPII protein, Sec23.. Nat Cell Biol.

[20] Cohen M, Stutz F, Dargemont C (2003). Deubiquitination, a new player in Golgi to endoplasmic reticulum retrograde transport.. J Biol Chem.

[21] Baxter BK, Abeliovich H, Zhang X, Stirling AG, Burlingame AL (2005). Atg19p ubiquitination and the cytoplasm to vacuole trafficking pathway in yeast.. J Biol Chem.

[22] Schmitz C, Kinner A, Kolling R (2005). The deubiquitinating enzyme Ubp1 affects sorting of the ATP-binding cassette-transporter Ste6 in the endocytic pathway.. Mol Biol Cell.

[23] Kee Y, Lyon N, Huibregtse JM (2005). The Rsp5 ubiquitin ligase is coupled to and antagonized by the Ubp2 deubiquitinating enzyme.. Embo J.

[24] Kee Y, Munoz W, Lyon N, Huibregtse JM (2006). The deubiquitinating enzyme Ubp2 modulates Rsp5-dependent Lys63-linked polyubiquitin conjugates in Saccharomyces cerevisiae.. J Biol Chem.

[25] Winzeler EA, Shoemaker DD, Astromoff A, Liang H, Anderson K (1999). Functional characterization of the S. cerevisiae genome by gene deletion and parallel analysis.. Science.

[26] Rigaut G, Shevchenko A, Rutz B, Wilm M, Mann M (1999). A generic protein purification method for protein complex characterization and proteome exploration.. Nat Biotechnol.

[27] Krogan NJ, Cagney G, Yu H, Zhong G, Guo X (2006). Global landscape of protein complexes in the yeast Saccharomyces cerevisiae.. Nature.

[28] Hein C, Springael JY, Volland C, Haguenauer-Tsapis R, Andre B (1995). NPl1, an essential yeast gene involved in induced degradation of Gap1 and Fur4 permeases, encodes the Rsp5 ubiquitin-protein ligase.. Mol Microbiol.

[29] Hoppe T, Matuschewski K, Rape M, Schlenker S, Ulrich HD (2000). Activation of a membrane-bound transcription factor by regulated ubiquitin/proteasome-dependent processing.. Cell.

[30] Dunn R, Klos DA, Adler AS, Hicke L (2004). The C2 domain of the Rsp5 ubiquitin ligase binds membrane phosphoinositides and directs ubiquitination of endosomal cargo.. J Cell Biol.

[31] Gwizdek C, Hobeika M, Kus B, Ossareh-Nazari B, Dargemont C (2005). The mRNA nuclear export factor Hpr1 is regulated by Rsp5-mediated ubiquitylation.. J Biol Chem.

[32] Chen HI, Einbond A, Kwak SJ, Linn H, Koepf E (1997). Characterization of the WW domain of human yes-associated protein and its polyproline-containing ligands.. J Biol Chem.

[33] Strano S, Munarriz E, Rossi M, Castagnoli L, Shaul Y (2001). Physical interaction with Yes-associated protein enhances p73 transcriptional activity.. J Biol Chem.

[34] Wang G, Yang J, Huibregtse JM (1999). Functional domains of the Rsp5 ubiquitin-protein ligase.. Mol Cell Biol.

[35] Katzmann DJ, Sarkar S, Chu T, Audhya A, Emr SD (2004). Multivesicular body sorting: ubiquitin ligase Rsp5 is required for the modification and sorting of carboxypeptidase S.. Mol Biol Cell.

[36] Kaminska J, Gajewska B, Hopper AK, Zoladek T (2002). Rsp5p, a new link between the actin cytoskeleton and endocytosis in the yeast Saccharomyces cerevisiae.. Mol Cell Biol.

[37] Li M, Chen D, Shiloh A, Luo J, Nikolaev AY (2002). Deubiquitination of p53 by HAUSP is an important pathway for p53 stabilization.. Nature.

[38] Longley DB, Harkin DP, Johnston PG (2003). 5-fluorouracil: mechanisms of action and clinical strategies.. Nat Rev Cancer.

[39] Jund R, Lacroute F (1970). Genetic and physiological aspects of resistance to 5-fluoropyrimidines in Saccharomyces cerevisiae.. J Bacteriol.

[40] Giaever G, Flaherty P, Kumm J, Proctor M, Nislow C (2004). Chemogenomic profiling: identifying the functional interactions of small molecules in yeast.. Proc Natl Acad Sci U S A.

[41] Swaminathan S, Amerik AY, Hochstrasser M (1999). The Doa4 deubiquitinating enzyme is required for ubiquitin homeostasis in yeast.. Mol Biol Cell.

[42] Dunn R, Hicke L (2001). Domains of the Rsp5 ubiquitin-protein ligase required for receptor-mediated and fluid-phase endocytosis.. Mol Biol Cell.

[43] Reneke JE, Blumer KJ, Courchesne WE, Thorner J (1988). The carboxy-terminal segment of the yeast alpha-factor receptor is a regulatory domain.. Cell.

[44] Schandel KA, Jenness DD (1994). Direct evidence for ligand-induced internalization of the yeast alpha-factor pheromone receptor.. Mol Cell Biol.

[45] Raths S, Rohrer J, Crausaz F, Riezman H (1993). end3 and end4: two mutants defective in receptor-mediated and fluid-phase endocytosis in Saccharomyces cerevisiae.. J Cell Biol.

[46] Bucking-Throm E, Duntze W, Hartwell LH, Manney TR (1973). Reversible arrest of haploid yeast cells in the initiation of DNA synthesis by a diffusible sex factor.. Exp Cell Res.

[47] Amerik AY, Hochstrasser M (2004). Mechanism and function of deubiquitinating enzymes.. Biochim Biophys Acta.

[48] Nijman SM, Luna-Vargas MP, Velds A, Brummelkamp TR, Dirac AM (2005). A genomic and functional inventory of deubiquitinating enzymes.. Cell.

[49] Ren J, Kee Y, Huibregtse JM, Piper RC (2007). Hse1, a Component of the Yeast Hrs-STAM Ubiquitin-sorting Complex, Associates with Ubiquitin Peptidases and a Ligase to Control Sorting Efficiency into Multivesicular Bodies.. Mol Biol Cell.

[50] Wang G, McCaffery JM, Wendland B, Dupre S, Haguenauer-Tsapis R (2001). Localization of the Rsp5p ubiquitin-protein ligase at multiple sites within the endocytic pathway.. Mol Cell Biol.

[51] Li M, Brooks CL, Kon N, Gu W (2004). A dynamic role of HAUSP in the p53-Mdm2 pathway.. Mol Cell.

[52] Mouchantaf R, Azakir BA, McPherson PS, Millard SM, Wood SA (2006). The ubiquitin ligase itch is auto-ubiquitylated in vivo and in vitro but is protected from degradation by interacting with the deubiquitylating enzyme FAM/USP9X.. J Biol Chem.

[53] Terrell J, Shih S, Dunn R, Hicke L (1998). A function for monoubiquitination in the internalization of a G protein-coupled receptor.. Mol Cell.

[54] Springael JY, Galan JM, Haguenauer-Tsapis R, Andre B (1999). NH4+-induced down-regulation of the Saccharomyces cerevisiae Gap1p permease involves its ubiquitination with lysine-63-linked chains.. J Cell Sci.

[55] Gitan RS, Eide DJ (2000). Zinc-regulated ubiquitin conjugation signals endocytosis of the yeast ZRT1 zinc transporter.. Biochem J.

[56] Stawiecka-Mirota M, Pokrzywa W, Morvan J, Zoladek T, Haguenauer-Tsapis R (2007). Targeting of Sna3p to the endosomal pathway depends on its interaction with Rsp5p and multivesicular body sorting on its ubiquitylation.. Traffic.

[57] Leon S, Erpapazoglou Z, Haguenauer-Tsapis R (2008). Ear1p and ssh4p are new adaptors of the ubiquitin ligase rsp5p for cargo ubiquitylation and sorting at multivesicular bodies.. Mol Biol Cell.

[58] Hicke L, Dunn R (2003). Regulation of membrane protein transport by ubiquitin and ubiquitin-binding proteins.. Annu Rev Cell Dev Biol.

[59] Swanson KA, Kang RS, Stamenova SD, Hicke L, Radhakrishnan I (2003). Solution structure of Vps27 UIM-ubiquitin complex important for endosomal sorting and receptor downregulation.. Embo J.

[60] Gietz D, St Jean A, Woods RA, Schiestl RH (1992). Improved method for high efficiency transformation of intact yeast cells.. Nucleic Acids Res.

[61] Soni R, Carmichael JP, Murray JA (1993). Parameters affecting lithium acetate-mediated transformation of Saccharomyces cerevisiae and development of a rapid and simplified procedure.. Curr Genet.

[62] Tong AH, Evangelista M, Parsons AB, Xu H, Bader GD (2001). Systematic genetic analysis with ordered arrays of yeast deletion mutants.. Science.

[63] Longtine MS, McKenzie A, Demarini DJ, Shah NG, Wach A (1998). Additional modules for versatile and economical PCR-based gene deletion and modification in Saccharomyces cerevisiae.. Yeast.

[64] Emili A, Schieltz DM, Yates JR, Hartwell LH (2001). Dynamic interaction of DNA damage checkpoint protein Rad53 with chromatin assembly factor Asf1.. Mol Cell.

[65] Marchal C, Dupre S, Urban-Grimal D (2002). Casein kinase I controls a late step in the endocytic trafficking of yeast uracil permease.. J Cell Sci.

[66] Hanna J, Leggett DS, Finley D (2003). Ubiquitin depletion as a key mediator of toxicity by translational inhibitors.. Mol Cell Biol.

[67] Stamenova SD, Dunn R, Adler AS, Hicke L (2004). The Rsp5 ubiquitin ligase binds to and ubiquitinates members of the yeast CIN85-endophilin complex, Sla1-Rvs167.. J Biol Chem.

[68] Brachmann CB, Davies A, Cost GJ, Caputo E, Li J (1998). Designer deletion strains derived from Saccharomyces cerevisiae S288C: a useful set of strains and plasmids for PCR-mediated gene disruption and other applications.. Yeast.

[69] Ghaemmaghami S, Huh WK, Bower K, Howson RW, Belle A (2003). Global analysis of protein expression in yeast.. Nature.

[70] Tong AH, Lesage G, Bader GD, Ding H, Xu H (2004). Global mapping of the yeast genetic interaction network.. Science.

[71] Wu X, Chang A, Sudol M, Hanes SD (2001). Genetic interactions between the ESS1 prolyl-isomerase and the RSP5 ubiquitin ligase reveal opposing effects on RNA polymerase II function.. Curr Genet.

