# Supplementary figures and images for: Interaction of the Deubiquitinating Enzyme Ubp2 and the E3 Ligase Rsp5 Is Required for Transporter/Receptor Sorting in the Multivesicular Body Pathway
Source: PLoS One. 2009 Jan 23;4(1):e4259. doi: 10.1371/journal.pone.0004259 (PMC2626285; doi:10.1371/journal.pone.0004259)

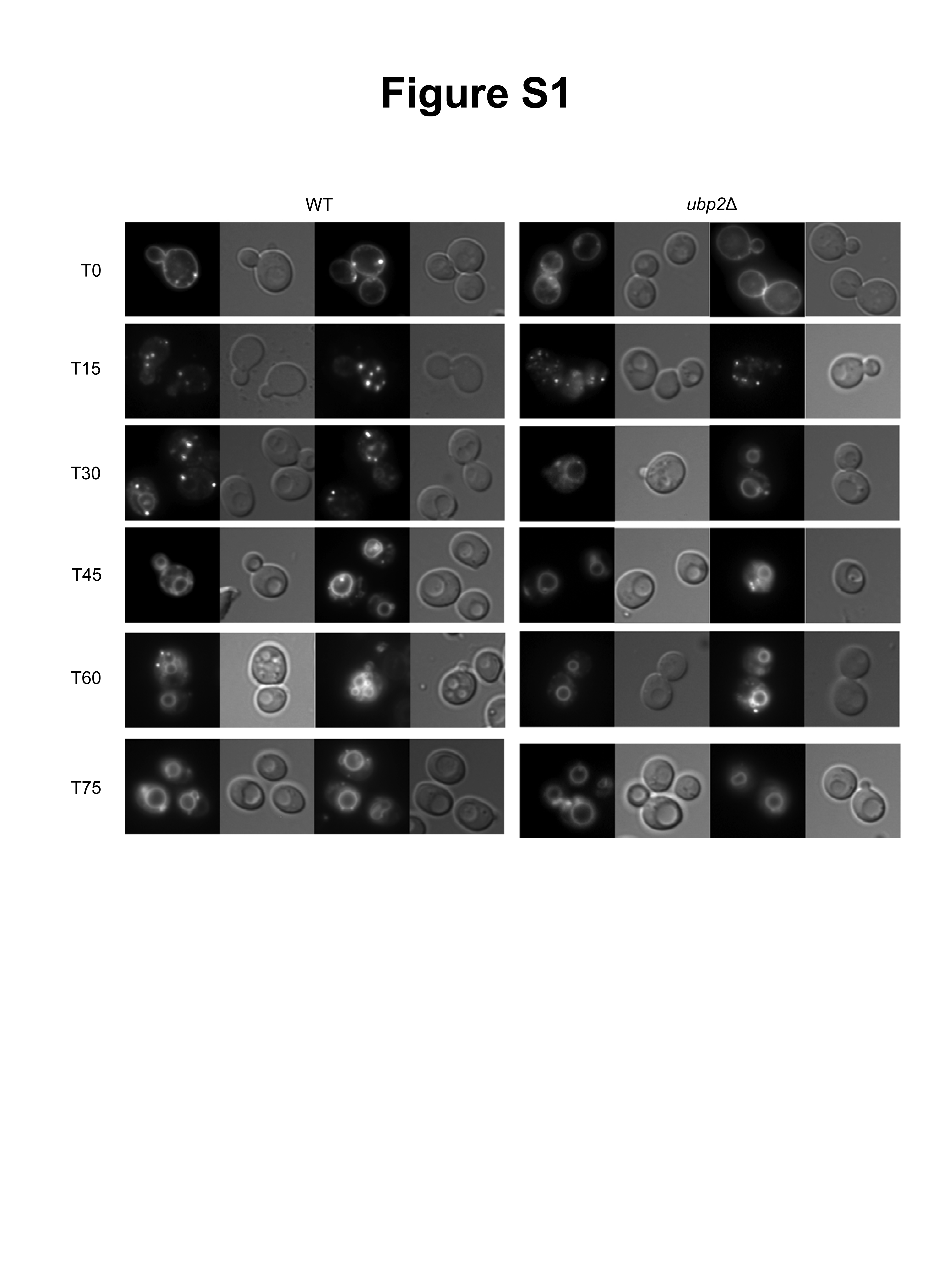

Supplement: Figure S1 — Endosome and Vacuolar morphology is normal in ubp2Δ cells. Cells were incubated with the lipid-binding fluorescent dye FM4-64 and washed. Endocytosis of the dye and endosome/vacuolar morphology were monitored by fluorescence microscopy and Nomarski optics at the indicated time points (min) after staining. (1.96 MB TIF) [file pone.0004259.s001.tif]

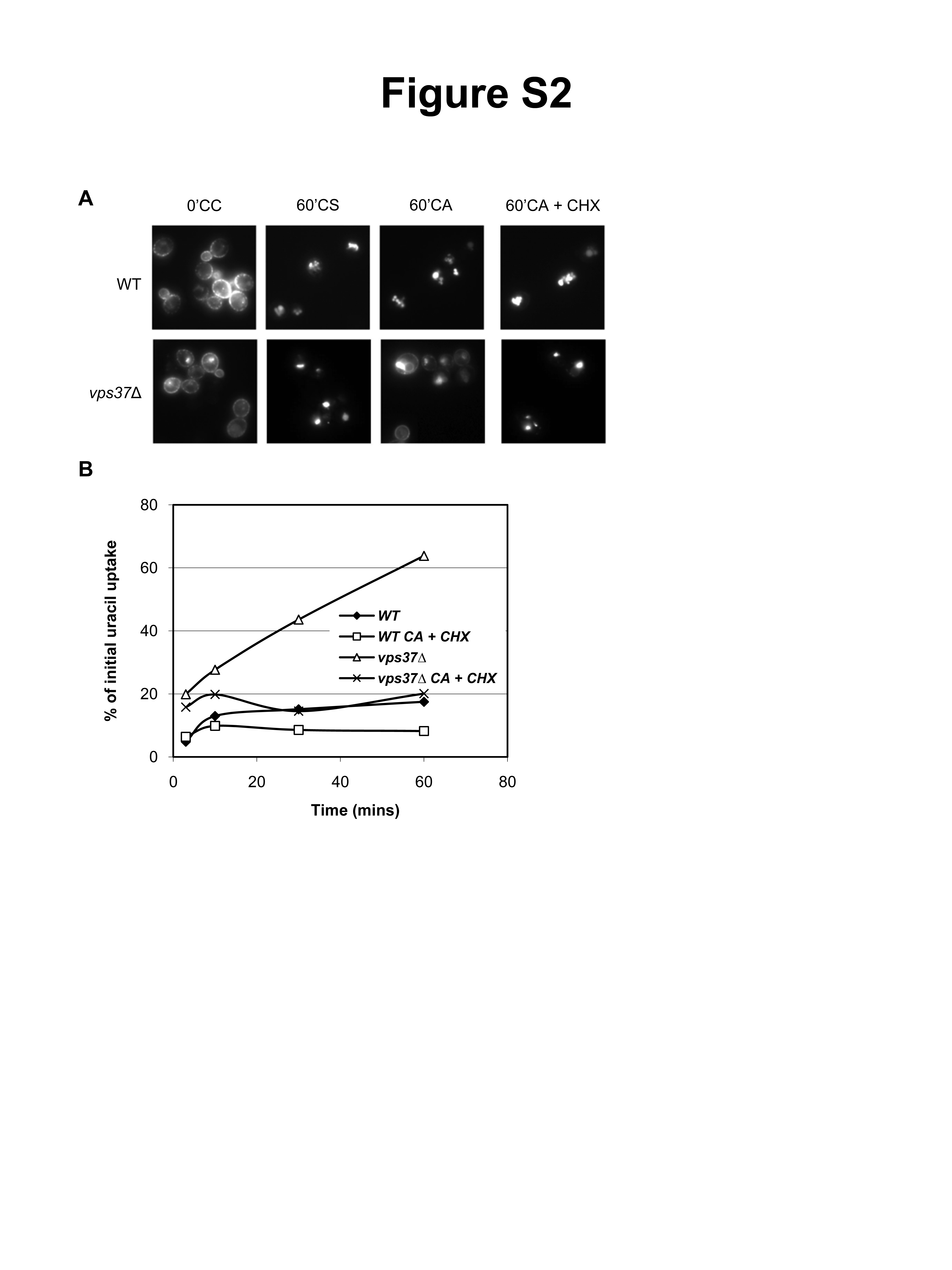

Supplement: Figure S2 — Recycling of Fur4-GFP cannot be detected in the presence of cycloheximide. WT and vps37Δ cells transformed with pFur4-GFP were cultured at 30°C and Fur4-GFP synthesis was induced for 90 min by adding galactose. Glucose was added to block Fur4-GFP synthesis (CC). 20 min later, cells were subjected to carbon starvation (CS) to trigger endocytosis of the permease for 60 minutes. Cultures were divided in two equal fractions. Glucose (CA) or glucose+cycloheximide (CA+CHX 0.1 mg/ml) was then added. (A) Cells were visualized by fluorescence microscopy at t = 0 (0'CC), after 60 minutes of carbon starvation (60'CS) and 60 minutes after the addition of carbon (60'CA) or carbon+cycloheximide (60'CA+CHX). Note that Fur4-GFP was efficiently internalized in all the strains upon CS. (B) Uracil uptake was measured 3, 10, 30 and 60 min after the addition of glucose. Results are expressed as a percentage of the initial uracil uptake measured immediately before carbon starvation, and plotted on a linear scale. (0.46 MB TIF) [file pone.0004259.s002.tif]
