# Supplementary material for: Interaction of the Deubiquitinating Enzyme Ubp2 and the E3 Ligase Rsp5 Is Required for Transporter/Receptor Sorting in the Multivesicular Body Pathway
Source: PLoS One. 2009 Jan 23;4(1):e4259. doi: 10.1371/journal.pone.0004259 (PMC2626285; doi:10.1371/journal.pone.0004259)
Supplement: Materials and Methods S1 — (0.04 MB DOC) [file pone.0004259.s003.doc]

SUPPLEMENTARY MATERIALS AND METHODS

*Strains and Plasmids*

Strains are WT (BY4741 *MATa* *his3*Δ*1* *leu2*Δ*0* *ura3*Δ*0* *met15*Δ*0*) [68] and *ubp2*Δ::*KanMX3* from the deletion consortium collection [25]. *vps37*Δ is *vps37*::HIS and was constructed as described in [63] with p*FUR4*-GFP transformed in.

*Microscopy*

Cells grown to exponential growth phase in YNB medium was concentrated by a factor of ten by centrifugation. Cells were viewed immediately, without fixation, under a fluorescence microscope (type BY61, Olympus, Tokyo, Japan) and images captured with a digital camera.

*FM4-64 Labelling*

1mL of culture was concentrated into 100µL, placed on ice, and incubated in the presence of 20µM FM4-64 (Molecular Probes) for 15 minutes. Cells were then washed twice with 1mL of cold medium, resuspended in 1mL of medium (T0) and incubated for the indicated times at room temperature. At each time point, 100µl of cells were diluted in cold water containing 10mM sodium azide, then concentrated into 10µL and examined for fluorescence.

*Recycling experiment*

Fur4-GFP was studied as previously described [12], except that the experiment was conducted at 30°C. Briefly, cells expressing Fur4-GFP at the plasma membrane were quickly filtered, washed and resuspended in minimal medium without a carbon source. Glucose was added 60 minutes after the start of carbon starvation. Redistribution of Fur4-GFP at the plasma membrane was followed by both fluorescence microscopy and uracil uptake measurements.
